# Management of Neonatal Aortic Thrombosis: Challenges and Outcomes With Systemic and Intra-arterial Thrombolysis

**DOI:** 10.7759/cureus.62280

**Published:** 2024-06-12

**Authors:** Ravi Reddy, Sagar Karotkar, Mahaveer S Lakra, Chaitanya Kumar Javvaji, Aditi Rawat, Sai Bhavani Manchineni

**Affiliations:** 1 Neonatology, Jawaharlal Nehru Medical College, Datta Meghe Institute of Higher Education and Research, Wardha, IND; 2 Pediatrics, Jawaharlal Nehru Medical College, Datta Meghe Institute of Higher Education and Research, Wardha, IND

**Keywords:** low-molecular-weight heparin, reteplase, tissue plasminogen activator (tpa), limb ischemia, atrial thrombosis

## Abstract

Neonatal aortic thrombosis, though rare, is associated with high mortality and is frequently linked to umbilical vessel catheterization, especially in smaller and critically ill infants due to their low levels of natural anticoagulants and increased prothrombotic activity. We report a case of a term neonate with abdominal aortic thrombosis and severe lower limb ischemia, presenting with respiratory distress requiring intubation and subsequent development of thrombosis by day 7. Initial anticoagulation with heparin proved insufficient, necessitating the use of reteplase and intra-arterial thrombolysis, which resulted in clinical improvement despite limited immediate success in Doppler studies. The patient was discharged on low-molecular-weight heparin against medical advice, highlighting the complexities and need for individualized management strategies in neonatal thromboembolism.

## Introduction

Although the occurrence of neonatal aortic thrombosis is uncommon, they have been documented in the literature [1] and are linked to a high death rate. The primary cause is the catheterization of the umbilical vessels. Smaller and sicker babies are more likely to experience catheterization-related difficulties because of factors such as low levels of natural anticoagulants and increased prothrombotic activity [2]. The therapy of arterial thrombus in newborns is still debatable. The thrombolytic drug that is most frequently utilized is recombinant tissue plasminogen activator [3]. Here, we describe our experience treating a newborn with this illness, an instance of abdominal aortic thrombosis in a patient treated for lower limb ischemia at an advanced stage.

Thromboembolism is a complex illness that involves a combination of acquired triggers, underlying medical conditions, and inherited predispositions. Beyond race/ethnicity, antithrombin III (AT), protein S (PS), and protein C (PC) deficiencies are known hereditary factors for thrombosis [4,3].

The longer the umbilical artery catheterization, the higher the risk of thrombosis. Even in the absence of genetic thrombophilias, newborns are most vulnerable to thrombosis during their formative years. A growing number of newborns and kids are being diagnosed with embolism as a side effect of congenital heart disease, cancer, sepsis, drug therapy-related events, as well as intravenous catheters. The risk of thromboembolism may increase due to recent developments in neonatal medicine.

Systemic anticoagulation with low-molecular-weight or unfractionated heparin is the standard treatment. Systemic thrombolytic therapy is indicated in cases of arterial occlusion, massive pulmonary embolism, pulmonary embolism not responding to heparin therapy, and hazard to organ or limb viability. The most often used thrombolytic medications in adult patients are streptokinase, urokinase, and recombinant tissue plasminogen activator (r-TPA). The Food and Drug Administration does not think urokinase is safe, and streptokinase is not advised for use in young patients due to the high prevalence of neutralizing anti-streptococcal antibodies. Consequently, r-TPA is the recommended thrombolytic medication in pediatrics [5]. That being said, not much r-TPA therapy has been administered for little prematures, especially during the first week of life.

## Case presentation

A 2.6 kg male neonate was delivered via caesarean section at term. The delivery was complicated by meconium-stained amniotic fluid. The infant cried immediately post-delivery but subsequently developed respiratory distress characterized by subcostal retractions, expiratory grunting, and tachypnea (respiratory rate of 70 breaths per minute). Continuous positive airway pressure (CPAP) was initiated, but due to worsening respiratory distress, the neonate was electively intubated and administered surfactant. On day 7, the attending neonatologist observed bluish discoloration of the right great toe and noted feeble femoral pulses bilaterally, more pronounced on the right than the left. Sepsis screen parameters were negative, the platelet count was .7 lakh/cumm, and the coagulation profile showed a prothrombin time (PT) of 11.9, an activated partial thromboplastin time (APTT) of 52.8, and an international normalized ratio (INR) of 1.0. Doppler studies of the lower limbs revealed a thrombus in the lower third of the abdominal aorta extending into the bilateral external iliac arteries. Anticoagulation therapy was initiated with a loading dose of intravenous (IV) heparin at 75 IU/kg, followed by a maintenance dose of 28 IU/kg/hour.

**Figure 1:**
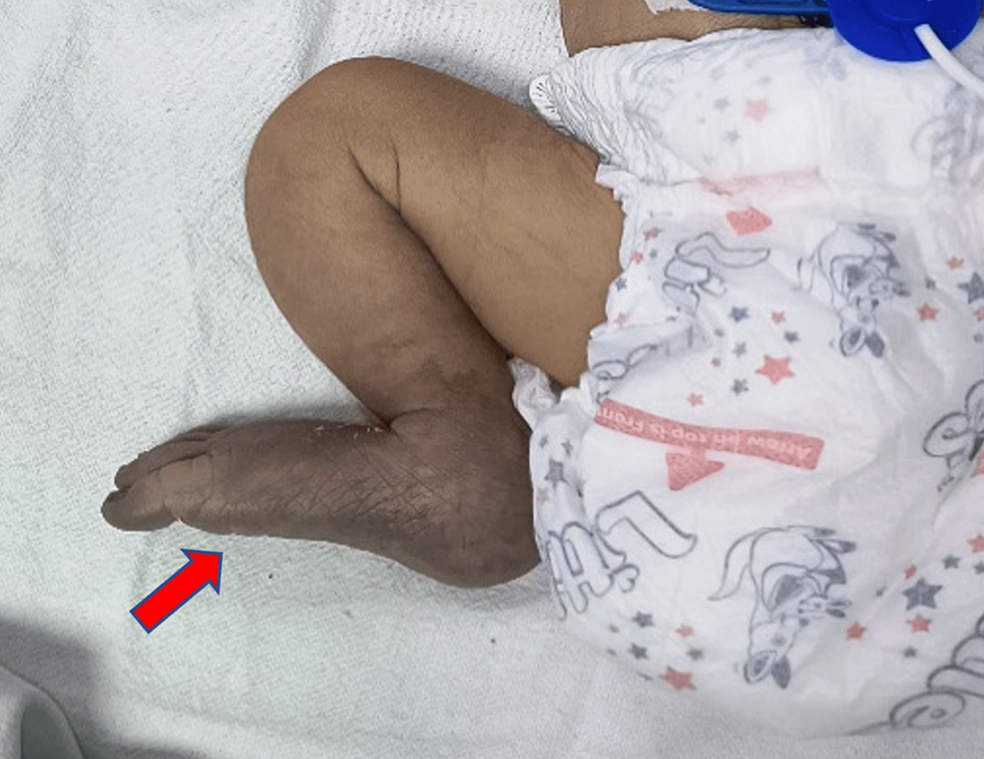
Clinical image at the time of admission showing clearly demarcated area of gangrene (red arrow)

The neonate was transferred to our facility on day 10 for further management. Upon admission, the patient, now 10 days old, was on bag-and-tube ventilation and subsequently transitioned to mechanical ventilation. Persistent cyanosis of the right lower extremity and absent femoral pulses (right greater than left) were noted (Figure 1). Laboratory evaluations were within normal limits.

Continuous heparin infusion was maintained, and follow-up Doppler studies confirmed the presence of thrombi; no progression nor reduction in the size of the thrombi was noted. A 2D echocardiogram revealed a thrombus in the left atrium, prompting a switch to low-molecular-weight heparin at 1.5 mg/kg/day. Tissue plasminogen activator (tPA) administration was planned based on clinical reassessment. Neurosonography was normal. Fresh frozen plasma was transfused to achieve adequate thrombolysis as supplementation with plasminogen before commencing thrombolytic therapy is recommended and random donor platelets were transfused to maintain a platelet count of 50-100×10^4^/microliter. Initial administration of reteplase did not yield a significant improvement in Doppler studies, although the left atrial thrombus resolved on echocardiography. Consequently, the reteplase dose was doubled and continued.

**Figure 2:**
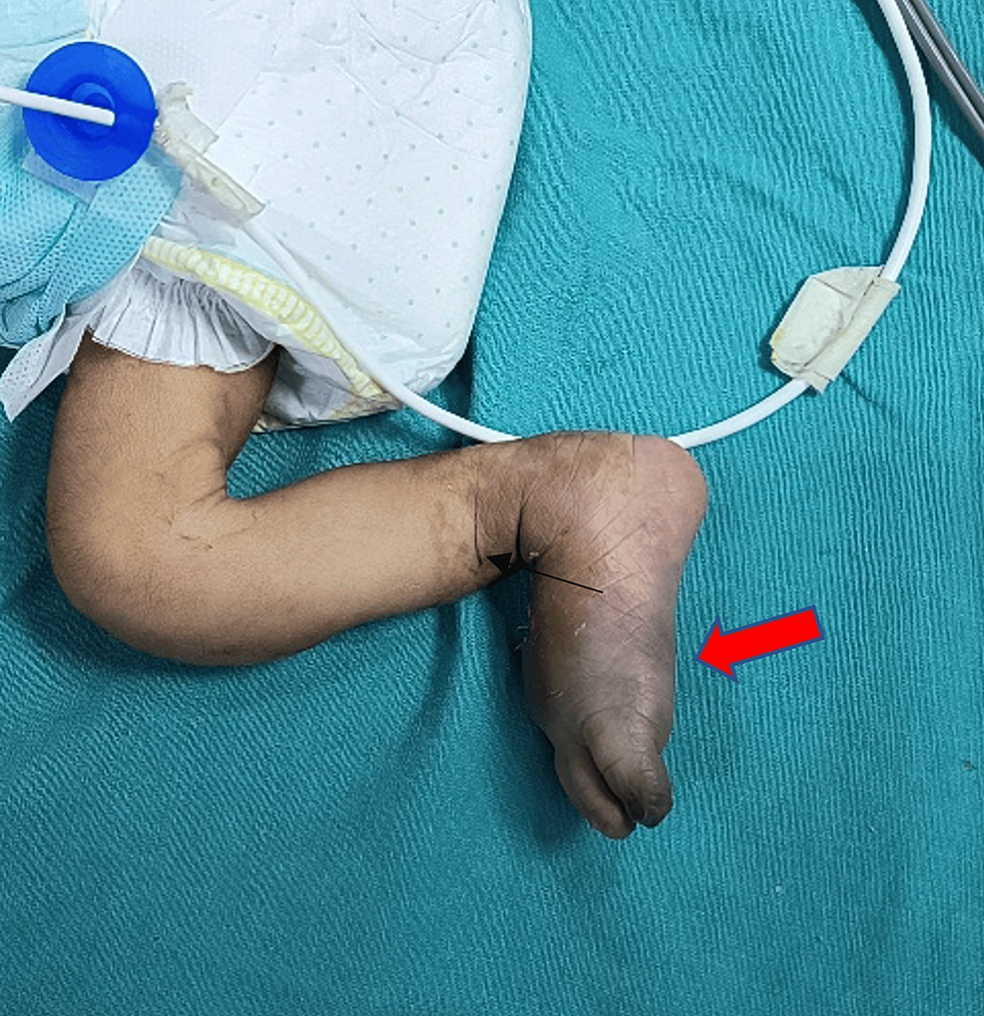
Clinical image after intra-arterial thrombolysis showing visible improvement in the right lower limb's color and also shriveled black toe as a consequence of gangrene (red arrow) (line of demarcation indicated (black arrow))

By day 3 post-admission, the patient was extubated and managed with CPAP and then gradually transitioned to heated humidified high-flow nasal cannula. Despite tPA therapy, there was no significant improvement in the peripheral pulses. Consultation with the interventional radiology team led to intra-arterial thrombolysis, which was uneventful and resulted in visible improvement in the right lower limb's color (Figure 2).

Post-thrombolysis, the infant received continuous heparin infusion for two days, followed by low-molecular-weight heparin for five additional days. Although further hospitalization was advised, the parents opted for discharge against medical advice.

## Discussion

Aortic thrombosis is associated with a number of risk factors, including coagulopathy, hypoxia, patent ductus arteriosus, polycythemia, maternal diabetes, and umbilical artery catheterization. Umbilical artery catheterization is the most common risk factor. An indwelling umbilical catheter is the single highest risk factor for aortic thrombosis; septicemia and dehydration are other risk factors that increase the chance of thrombosis [6]. 

Additional research revealed that in cases of symptomatic newborn thrombosis, the venous and arterial systems are equally implicated. When there are significant or progressive ischemia alterations, early surgical surgery is recommended. More frequently, there has been a shift toward autoamputation or spontaneous slough or toward surgery and restricted amputation [6-8].

For neonates who are diagnosed with aortic thrombosis, the use of TPA for thrombolysis should be as per protocol. Usually, it is continuous infusion for six hours, or sometimes prolonged usage is required to restore limb perfusion and clot lysis. Neonates presenting with impending tissue loss or significant end-organ damage may suffer permanent ischemic injury during the prolonged period. This period of relative ischemia can be tolerated by patients with small thrombus.

To preserve limb viability, the r-TPA infusion dose in the current case study was increased from 0.1 mg/kg/hour to 0.4 mg/kg/hour. It's possible that more FFP treatment will help to dissolve the clot. When managing newborns with vascular blockages that pose a risk to their lives or limbs, safe and effective thrombolytic treatment is crucial. Four cases of lower limb gangrene in newborns were treated by Kothari et al.; all four had bilateral gangrene, requiring amputation [9], and in two of them, there was no known cause. Additionally, Nagai et al. reported two cases of bilateral lower limb intrauterine gangrene aggravated by twin-to-twin transfusion syndrome that required amputations below the knee [10].

## Conclusions

This case report highlights the challenges and complexities in managing neonatal aortic thrombosis, particularly in the context of severe lower limb ischemia. The use of reteplase and intra-arterial thrombolysis demonstrated potential efficacy in resolving thrombotic events when conventional anticoagulation therapy with heparin was insufficient. Despite the initial limited success observed in Doppler studies, clinical improvement was noted, emphasizing the importance of aggressive and timely intervention in such critical cases. This case underscores the need for individualized treatment approaches and further research to establish standardized protocols for managing thromboembolic events in neonates.

## References

[1] Hamilton RM, Penkoske PA, Byrne P, Duncan NF (1988). Spontaneous aortic thrombosis in a neonate presenting as coarctation. Ann Thorac Surg.

[2] Richardson MW, Allen GA, Monahan PE (2002). Thrombosis in children: current perspective and distinct challenges. Thromb Haemost.

[3] Boo NY, Wong NC, Zulkifli SS, Lye MS (1999). Risk factors associated with umbilical vascular catheter-associated thrombosis in newborn infants. J Paediatr Child Health.

[4] Ichiyama M, Ohga S, Ochiai M (2016). Age-specific onset and distribution of the natural anticoagulant deficiency in pediatric thromboembolism. Pediatr Res.

[5] Oppenheimer EH, Esterly JR (1965). Thrombosis in the newborn: comparison between infants of diabetic and nondiabetic mothers. J Pediatr.

[6] Ibrahim H, Krouskop R, Jeroudi M, McCulloch C, Parupia H, Dhanireddy R (2001). Venous gangrene of lower extremities and Staphylococcus aureus sepsis. J Perinatol.

[7] Kanzenbach TL, Dexter WW (1999). Cold injuries. Protecting your patients from the dangers of hypothermia and frostbite. Postgrad Med.

[8] Letts M, Blastorah B, al-Azzam S (1997). Neonatal gangrene of the extremities. J Pediatr Orthop.

[9] Kothari PR, Gupta A, Kulkarni B (2005). Neonatal lower extremity gangrene. Indian Pediatr.

[10] Nagai MK, Littleton AG, Gabos PG (2007). Intrauterine gangrene of the lower extremity in the newborn: a report of two cases. J Pediatr Orthop.

